# Stressful Factors in the Working Environment, Lack of Adequate Sleep, and Musculoskeletal Pain among Nursing Unit Managers

**DOI:** 10.3390/ijerph17020673

**Published:** 2020-01-20

**Authors:** Hjördís Sigursteinsdóttir, Hafdís Skúladóttir, Thórey Agnarsdóttir, Sigrídur Halldórsdóttir

**Affiliations:** 1 School of Business and Science, University of Akureyri, Nordurslod 2, 600 Akureyri, Iceland; 2 School of Health Sciences, University of Akureyri, Nordurslod 2, 600 Akureyri, Iceland; hafdis@unak.is (H.S.); sigridur@unak.is (S.H.); 3 Environmental and Public Health Authority, Furuvellir 1, 600 Akureyri, Iceland

**Keywords:** working environment, middle managers, nursing unit managers, musculoskeletal pain, stress factors, adequate sleep

## Abstract

Background: Middle managers have not received enough attention within the healthcare field, and little is known how stressful factors in their work environment coupled with a lack of adequate sleep are related to musculoskeletal pain. The aim of this study was to examine the correlation between stressful factors in the work environment, lack of adequate sleep, and pain/discomfort in three body areas. Methods: Questionnaire was sent electronically to all female nursing unit managers (NUM) in Iceland through the outcome-survey system. The response rate was 80.9%. Results: NUM who had high pain/discomfort in the neck area also had very high pain/discomfort in the shoulder area and pain in the lower back. The results also revealed positive a medium-strong correlation between mental and physical exhaustion at the end of the workday and musculoskeletal pain. Stress in daily work, mental strain at work, and being under time-pressures had hardly any correlation with pain/discomfort in the three body parts. Adequate sleep had a significant negative correlation with all stressful factors in the work environment and all three body parts under review. Conclusion: The results will hopefully lead to a better consideration of stressful factors in the work environment, sleep, and musculoskeletal pain in middle managers.

## 1. Introduction

Middle managers are in demanding roles and often find themselves in difficult positions. They play a crucial role in many institutions, and their demanding jobs are characterized by significant workloads and stress, along with continual time constraints and considerable communication demands [1,2,3,4,5]. The middle-management work environment has changed in recent years and has become increasingly complex and demanding. Although middle managers are trying to maintain high-quality standards within a tight financial framework, they also struggle with meeting employees’ needs and promoting greater customer satisfaction [6,7]. Middle managers are managers who have received little attention in both management studies and in discussions about the role of managers, especially within the health services [8]. To meet the modern needs of employees and patients, the role of middle managers has expanded and is one of the most demanding roles in healthcare [5]. However, we know very little about the effect of this challenging work on musculoskeletal pain or sleep. The purpose of this study was to examine the correlation between stressful factors in the work environment, lack of adequate sleep, and pain/discomfort in the neck/neck area, shoulder/shoulder area, and lower back among Icelandic nursing unit managers (NUM), a group of healthcare workers who do not work the night shift.

Work-related stress occurs when there is a discrepancy between job requirements, abilities, needs, and characteristics [9]. A systematic review of 2426 studies was conducted regarding the relationship between work-related psychosocial risk factors and stress-related problems and seven prospective studies were ultimately used. The systematic review revealed that high demands at work, minimal control of working conditions, little support from colleagues and superiors, and a seriously compromised employee sense of righteousness cause stress-related problems [10], resulting in poor health and reduced working capacity [11]. A more recent study focusing on healthcare professionals reported similar results, but the researchers concluded that recognition was the most crucial factor in reducing work-related stress [12]. When individuals must deal with stressful situations without real opportunities to influence them, it can lead to persistent stress [13].

According to a 2006 Icelandic government employee survey, the workload was highest at health institutions, and 68% of healthcare workers considered themselves almost always or always under a heavy workload. The results also revealed that healthcare workers experienced the highest stress levels [14]. Although, the results of the Icelandic survey are from 2006, they are in line with other more recent studies showing that stress is a significant problem in the health services [15,16], especially among nursing unit managers [16]. Nursing unit managers play an essential role in the workplace. Their actions can affect the well-being of nurses in their unit [17,18,19] and they are responsible for the quality of service provided [5,20].

In the study by Larsman et al. [21], where the same group was followed for up to four years (N = 1133), it was found that high demands at work directly cause work-related stress. This result is in line with a summary and analysis of 50 studies by Lang et al. [22]. Mental stress causes muscle tension and is associated with musculoskeletal problems, such as pain [23,24,25]—especially neck and shoulder pain [26]. Risk factors for neck pain include having a demanding job where people do not have much scope for activities [27]. A poor psychosocial work environment has been linked to musculoskeletal disorders [28,29] and is often related to time constraints [30].

Chronic musculoskeletal pain has a significant impact on quality of life, work ability, and activity, and is a known cause of work-related illness [31,32]. The results of the Fifth European Working Conditions Survey showed that 45% of women and 41% of men experienced neck, shoulder, and upper-back pain during the 12 months before the survey [33]. In a systematic review and best-evidence synthesis where 109 studies of workers from 1980 to 2006 were examined, neck pain occurred in 27.1–47.8% of the cases [27]. Frequent pain in the musculoskeletal system has a negative impact on work ability [34], and if the pain is present in other areas of the body, the ability to work decreases [25,35,36].

The relationship among stress, sleep problems, and musculoskeletal pain has been shown [30], but given the importance of sleep, it is strange how often lack of adequate sleep is omitted when assessing the general health of individuals—especially when assessing their musculoskeletal system [37]. High demands at work and a high workload are known to cause sleep disturbances [38,39], but stressful factors in the work environment are the most common causes of sleep problems [40]. Stress negatively affects both the length and quality of sleep [41]. Lack of adequate sleep (<7 h) increases the likelihood of fatigue [42] and colds [43] and increases the risk of mental health problems [44].

There are surprisingly few studies on the relationship among stress, lack of adequate sleep, and musculoskeletal pain, and none were found for nursing unit managers. In this study, we analyzed musculoskeletal pain/discomfort in the neck and neck area, the shoulder and shoulder area, and the lower back, stressful factors in the work environment, and adequate sleep among Icelandic nursing unit managers, along with the correlation between these three factors. The research questions were: (1) On a scale of 1–10, how severe is the pain/discomfort in the neck and neck area, shoulder and shoulder area, and lower back among the nursing unit managers, and does the pain/discomfort differ by age, seniority in a management position, number of full-time nursing positions, or workday length? (2) How often do nursing unit managers experience stress at work, mental strain, time pressures, and physical and mental exhaustion, and do their experiences differ by age, seniority in a management position, number of years in a full-time position in nursing, or workday length? (3) Do nursing unit managers get adequate sleep each week, and does it vary by age, seniority in a management position, number of full-time nursing positions, or workday length? (4) What is the correlation between the pain/discomfort in the neck and neck area, shoulder and shoulder area, and lower back, stressful factors in the work environment, and lack of adequate sleep?

## 2. Materials and Methods

Data were collected using pretested questionnaires sent electronically to participants in this descriptive cross-sectional study. The National Bioethics Committee in Iceland approved the study.

### 2.1. Online Survey and Sample

An online survey was given to NUM in all hospitals in Iceland. A total of 141 individuals, 136 women and four men, carried the ‘nursing unit manager’ job title at the time of the study. Due to the small number of male NUM, they were excluded from the study. The questionnaire was sent electronically from the outcome-survey system to 136 female NUM with an informational letter emphasizing that the answers would not be traced back to individual participants and full data confidentiality would be ensured. After three email reminders, 110 NUM had answered the questionnaire partially or completely, resulting in a response rate of 80.9%.

### 2.2. Survey Questions

The survey devised for this study was based on two well-tested questionnaires from the Research Institute of Nursing and the Occupational Safety and Health Administration in Iceland. The Independent variables were age (31–40 years, 41–50 years, 51–60 years, and 61 years and older), seniority in a managerial position (five years or less, 6–10 years, 11–15 years, and 16 years or longer), number of full-time nursing positions (1–6, 7–10, 11–15, and 16 or more), length of work day (8 h and fewer, 9 h, 10 h, and 11 h), stressful factors, and an adequate night’s sleep. In Iceland, nursing unit managers do not work on nightshifts or weekends and the workday does not vary with start time. Therefore, we only measured the length of the workday. Stressful factors were measured with five questions: (1) ‘Do you experience stress in your daily work?’ (2) ‘Do you experience mental strain at work?’ (3) ‘Are you under a lot of time-pressure at work?’ (4) ‘Are you physically exhausted at the end of the workday?’ and (5) ‘Are you mentally exhausted at the end of the workday?’ There were five response options: 1 = very seldom or never, 2 = rather seldom, 3 = sometimes, 4 = rather often, and 5 = very often or always. These three questions were combined into one variable called *stressful factors* (Cronbach’s Alpha = 0.847). An adequate night’s sleep was measured with one question: ‘Do you get enough sleep?’ There were three response options for this question: 1 = yes, 5–7 nights of the week, 2 = yes, 3–4 nights of the week, 3 = no, only 2 nights of the week or less.

Three dependent variables were used to obtain a greater understanding of musculoskeletal pain among the NUM: (1) ‘How much discomfort/pain have you had in the neck or neck area on a scale of 1–10 in the past six months?’; (2) ‘How much discomfort/pain have you had in your shoulders or shoulder area on a scale of 1–10 in the past six months?’; and (3) ‘How much discomfort/pain have you had in the lower back on a scale of 1–10 in the past six months?’, where 1 = mild physical discomfort and 10 = most enormous pain imaginable.

### 2.3. Statistical Processing

Descriptive and exploratory data analyses were used to present the results for the examined variables (mean, standard deviation, and ratio). One-way ANOVA was performed to investigate differences in musculoskeletal pain/discomfort by age, seniority in a managerial position, number of full-time positions in nursing, and length of workday. The chi-square test was used to examine differences between stressful factors in the work environment and an adequate night’s sleep, age, seniority in a managerial position, number of a full-time positions in nursing, and workday length. 

The effect size was calculated with Cramer’s V. Spearman’s Rho was used to evaluate the relationship among pain/discomfort in the neck and neck area, shoulder and shoulder area, and lower back and stressful factors in the working environment and lack of adequate sleep. The strength of the relationship was determined according to Cohen’s (1988, pp. 79–81) guidelines, where r = 0.10 to 0.29 is small, r = 0.30 to 0.49 is medium and r = 0.50 to 1.0 is large.

Statistical tests were conducted at a 5% level of significance, and data analyses were obtained from SPSS version 22.0 (IBM, Armonk, NY, USA).

## 3. Results

### 3.1. Characteristics of the Study Sample

Over half, or 56 out of 110, of the NUM were aged 51–60 (51%). About 38% were aged 41–50, 11% were aged 31–40, and 4% were older than 60. Proportionally, most NUM had been in management positions for 16 years or more (32%), and about a quarter had been in these positions for 11–15 years. Over 26% had been in management positions for five years or less, and about 18% had held these positions for six to 10 years.

When asked about the number of full-time equivalent nursing positions in a ward, it was determined that the proportion was relatively equal among all groups. Just under a quarter worked at a ward with one to six full-time equivalent positions, another quarter worked at a ward with seven to 10 full-time equivalent positions, another quarter worked at a ward with 11–15 full-time equivalent positions, and just over a quarter worked at a ward with 16 or more full-time equivalent positions.

About 41% of the NUM worked eight hours or less a day, 43% worked nine hours a day, 11 worked 10 hours, a day, and 5% worked 11 hours or more each day.

### 3.2. Musculoskeletal Pain among the Nursing Unit Managers

Only six out of 110 NUM responded that they had no neck, shoulder, or back discomfort/pain. However, 52% (±9.3%) had discomfort/pain in all three of these body areas. About 30% (±8.6%) of the NUM had discomfort/pain in two body areas, and 11% (±5.8%) had discomfort/pain in one of these body areas.

Table 1 presents the severity of discomfort/pain in the neck and/or neck area, the shoulder and/or shoulder area, and the lower back on a scale of 1–10 in the past six months. The NUM usually had discomfort/pain in the shoulders and/or shoulder area (83%), and the average discomfort intensity was highest in that body area (*M* = 4.8; *SD* = 2.4). The next most common item was that the NUM had discomfort/pain in the neck and/or neck area (81%), and the mean intensity of the discomfort/pain was 4.7 (*SD* = 2.4). About 72% of the NUM had lower back discomfort/pain, and the average discomfort intensity was 4.6 (*SD* = 2.6).

There were no statistical differences in the mean intensity of discomfort/pain in any of these three body areas by age, seniority in a management position, number of full-time positions at nursing, or workday length.

### 3.3. Stressful Factors and an Adequate Night’s Sleep

As shown in Table 2, about 43% (±9.3%) of the NUM reported being often or always under a lot of time pressure at work, nearly 34% (±8.9%) were often or always mentally exhausted at the end of the workday and 32% (±8.7%) often or always experienced mental strain at work. Conversely, about 59% (±9.2%) reported sometimes experiencing stress in their daily work, and 37% (±9.0%) of the NUM reported that they seldom or never felt physically exhausted at the end of the workday.

The chi-square test only revealed a significant difference between how often the NUM are under a lot of time pressure at work and the length of the workday (χ*^2^*_(12,107)_ = 35.2, *p* < 0.05), indicating a strong association between workday length and time pressure at work (Cramer’s V = 0.33).

Table 3 displays the results for an adequate night’s sleep by age, seniority in a managerial position, number of full-time positions in nursing, and workday length. Just over half of the NUM received enough sleep each week/five to seven nights per week (56%, ±9.3%). About 12% (±6.1%) of the NUM rarely had an adequate night’s sleep/one to two nights per week. The results revealed no statistical differences among an adequate night’s sleep and age, seniority in a management position, number of full-time positions in nursing, and workday length.

### 3.4. The Correlation of Stressful Factors, an Adequate Night’s Sleep, and Musculoskeletal Pain

Table 4 shows the results of the correlation among the pain/discomfort in the neck and neck area, shoulder and shoulder area, and lower back, stressful factors in the working environment, and a lack of adequate sleep. There was a strong positive correlation between pain in the neck and neck area and pain in the shoulder and shoulder area (*r* = 0.71), pain in the shoulder and shoulder area and pain in the lower back (*r* = 0.55), experiencing stress in daily work and experiencing mental strain at work (*r* = 0.63), experiencing stress in daily work and experiencing a lot of time-pressure at work (*r* = 0.52), experiencing mental strain at work and mental exhaustion at the end of the workday (*r* = 0.63), being under a lot of time pressure at work and mental exhaustion at the end of the workday (*r* = 0.52), and physical exhaustion at the end of the workday and mental exhaustion at the end of the workday (*r* = 0.53). The results revealed no statistical correlation between pain in the neck and neck area and experiencing stress in daily work (*r* = 0.13) and being under a lot of time pressure (*r* = 0.14), Still, there was a negative a medium-strong correlation with adequate sleep (*r* = −0.31), indicating that stress in daily work and being under time pressure does not explain pain/discomfort in the neck and neck area. Pain in the shoulder and shoulder area only correlated significantly with two of five stressful factors in the environment: Physical (*r* = 0.46) and mental exhaustion at the end of the workday (*r* = 0.30). There was also a negative medium-strong correlation between pain in the shoulder and shoulder area and adequate sleep (*r* = −0.33). Pain in the lower back only correlated statistically with physical exhaustion at the end of the workday (*r* = 0.46) and adequate sleep (*r* = −0.28).

## 4. Discussion

The aim of the study was to examine the correlation between factors in the work environment, lack of adequate sleep, and pain/discomfort in the neck/neck area, shoulder/shoulder area, and lower back. The results show that the NUM who had significant pain/discomfort in the neck and neck area also had very significant pain/discomfort in the shoulder and shoulder area and pain in the the lower back. The results also show a positive medium-strong correlation between mental and physical exhaustion at the end of the workday and musculoskeletal pain and show that stressful factors are also related to sleep problems. Three stressful factors in the work environment, experiencing stress in daily work, experiencing mental strain at work, and being under time pressure, had very little to no correlation with pain/discomfort in the three body parts under review. Adequate sleep had a significant negative correlation with all stressful factors in the work environment and all three body parts under review.

It is known that NUM are under enormous strain [45]. Almost half of the NUM in this study were often or always under a lot of time-pressure at work. Van Bogaert et al. [46] found that one in six NUM [N = 365] were emotionally exhausted or very tired after each workday. In this study, almost 34% of the participants were often or always mentally exhausted after the workday, and about a fifth of them were often or always physically exhausted. Having an excessive role overload causes significant stress for NUM [47], and they are more likely to report poorer physical health if their work demands are high and they experience a lack of support [48].

The study reveals correlation between mental and physical exhaustion at the end of the workday and musculoskeletal pain and shows that stressful factors are also related to sleep problems. However, it may be that these relationships are interactive to some extent, and mental and physical exhaustion at the end of the workday coupled with a lack of adequate sleep can cause musculoskeletal pain. Perhaps there is an opposite causal link. It can be exhaustive to be in a demanding job if one has pain, as pain can cause mental strain and affect one’s ability to work [11]. Then, pain can cause the individual to experience more exhaustion than those who do not have pain. It is often more difficult for people in pain to quickly execute tasks [49]. The same can be said of the causal relationship between pain and a lack of adequate sleep. People who suffer from pain may find it harder to sleep because of the pain [50].

Middle managers, such as NUM, take on multiple demands. According to Hermkens [1], the demands put on them can come from their bosses, subordinates, and other managers. The responsibilities of NUM are considerable and the tasks are varied. Despite a long workday, they are usually not able to complete all their assigned tasks [51]. Stress increases when it is not possible to complete the day’s work and the pile of waiting tasks steadily increases [51]. It should be considered that musculoskeletal pain has a negative effect on work capacity [34]. Since the pain is present in more areas of the body, the ability to work decreases [25,35,36].

Persistent workloads without rest can lead to persistent stress [13], and the relaxation that sleep should provide will not happen [52]. It is important for NUM to recognize stress indicators, like mild tension and mental exhaustion [34,53,54], and respond immediately with efforts to reduce stress and its effects, such as seeking increased support [10,45]. Furthermore, endurance training during the daytime can contribute to a better night’s sleep [55]. However, further research needs to be conducted on how to lower the stress of NUM to reduce their musculoskeletal pain and improve their sleep.

The main strength of this study is the novelty of the material and the participation level (81%). It was also advantageous to select only those in the sample who had the title of ‘nursing unit manager’ (no other occupations). This was done to ensure that all participants in the study did the same job. However, this can also be a limitation, as individuals in the same position with a different job title were not included. This study also gives valuable insights to better understanding the role of sleep among nursing unit managers who do not work night shifts. It can been seen as a weakness that only one question was used to measure adequate sleep. Since the nursing unit managers only work during the day and not on weekends and the workday does not vary with start time, self-evaluation of adequate sleep per week was an appropriate measurement at the time. It may be helpful in future research to include more questions about the sleep. It would also be useful in future studies to evaluated the presence of concomitant pathologies both in the osteoarticular apparatus (discus protrusions, previous dislocations of the shoulder, possible traumas suffered), and neuropsychiatric (anxiety, depression, sleep disorders).

This study’s main limitation is the number of NUM in Iceland, which restricts analytical models. Another limitation of the study is that it is difficult to compare differences between institutions. Most participants worked at Landspitali University Hospital (LUH), the largest hospital in Iceland, and such a comparison would not be significant. Furthermore, NUM may be under more strain than various other public service middle managers. Surveys conducted in 2006 on the work environment of Icelandic government employees revealed that the workload was highest at healthcare institutions, and 68% of healthcare workers were always stressed and under a heavy workload. This may indicate that NUM have a greater workload than many other public service middle managers. Indeed, research results indicate that stress is a major problem in the health service sector [15], especially in NUM [16].

It is worth investigating the relationship among stressful factors in the working environment, sleep, and musculoskeletal pain in several groups of middle managers who only work dayshifts. It would also be interesting to examine their health and well-being about other factors such as heart disease [56] and other lifestyle diseases, as they have been shown to be somewhat stress-related [57]. As Cannizzaro et al. [58] pointed out that in the professional work environment, there are nonspecific psychosocial and environmental risks that can determine different organic and behavioural disorder, therefore highlighting the demand for further research to detect the specific neuroendocrine and genetic factors that subtend and predict vulnerability to the work-related stress response.

In their work, NUM need to deal with a variety of tasks, both mentally and physically, and how they address such situations varies considerably. Duffield et al. [20] pointed out that NUM have important jobs within hospitals, and much depends on the quality of their service. Therefore, the nursing unit’s working environment must be cared for to enable NUM to perform their jobs in the best possible way. Indeed, Gianfermi and Buchholz [6] pointed out that the nursing unit’s work environment has changed much in recent years, and greater demands are placed on NUM in terms of both financial restraints and greater service. Lee et al. [59] and Shirey et al. [45] also debated the changing work environment involving the nursing unit, human resources, and optimization measures, which has led to an increased workload in the nursing unit. It may be more difficult for the nursing director to meet these increased requirements if the needed resources are unavailable.

From their study, McCallin and Frankson [60] concluded that the work of the NUM is complex, multifaceted, and challenging. Therefore, support for them is important. Lee and Cummings [61] also concluded that support from superiors is central to the nursing department’s job satisfaction and aids in workload management. It appears that we need to reduce the workload of NUM due to their known stress and heavy workload [47]. We agree with Van Bogaert et al. [46] on finding effective ways to support NUM and the teams they manage. It is best to consider both the individual and the organization when addressing musculoskeletal pain, as such measures are most successful when all parties are involved [29].

In future studies, it would be interesting to know how factors like autonomy and control at work also impact the relationships of stressful factors in the working environment, lack of adequate sleep, and musculoskeletal pain. Some additional details about shift length and timing and sleep would also be useful in subsequent studies, as well as some longitudinal or time series analyses. It would also be useful for future study to take into consideration the body weight and the body mass index in future samples.

## 5. Conclusions

The results showed that NUM who had high pain/discomfort in the neck/neck area also had very high pain/discomfort in the shoulder/shoulder area and pain in the lower back. The results also revealed a positive medium-strong correlation between mental and physical exhaustion at the end of the workday and musculoskeletal pain. Furthermore, adequate sleep had a significant negative correlation with all stressful factors in the work environment and all three body parts under review. From these findings, it can be concluded that being a NUM is a demanding job, which affects both mental and physical health of the NUM and can have negative influence on musculoskeletal pain and adequate sleep. Hopefully, the results of this study will lead to further studies on the relationship between stressful factors in work environment of middle management, musculoskeletal pain, and adequate sleep. It is important to promote a good healthy work environment, both for the health and well-being of the middle managements and the organizations.

## Figures and Tables

**Table 1:** The severity of discomfort/pain in the neck and/or neck area, the shoulder and/or shoulder area, and the lower back on a scale of 1–10 in the past six months.

	Neck or Neck Area	Shoulder and Shoulder Area	Lower Back
	Mean (SD)	Mean (SD)	Mean (SD)
Total	4.73 (2.4)	4.80 (2.4)	4.58 (2.6)
Age (years):			
31–40	5.17 (2.6)	4.40 (2.5)	3.73 (2.5)
41–50	4.35 (2.2)	4.44 (2.6)	4.57 (2.6)
51–60	4.94 (2.4)	5.17 (2.3)	4.84 (2.8)
>60	4.00 (2.6)	3.75 (2.2)	4.00 (1.4)
Seniority in a managerial position (years):			
<6	4.38 (2.5)	4.35 (2.5)	3.91 (2.5)
6–10	4.75 (2.4)	4.28 (2.8)	4.82 (2.6)
11–15	5.04 (2.2)	5.08 (2.2)	4.90 (2.9)
>15	4.77 (2.4)	5.18 (2.3)	4.71 (2.6)
Number of full-time positions in nursing:			
1–6	4.86 (2.2)	5.09 (2.5)	5.37 (2.7)
7–10	5.61 (2.5)	5.29 (2.5)	4.57 (2.7)
11–15	4.19 (2.2)	4.41 (2.4)	3.82 (2.7)
>15	4.54 (2.5)	4.77 (2.5)	4.52 (2.3)
Working hours per day			
≤8	4.51 (2.4)	4.73 (2.6)	4.85 (2.6)
9	4.76 (2.3)	4.81 (2.3)	4.53 (2.8)
10	4.90 (2.5)	5.10 (2.2)	3.75 (2.1)
11	5.60 (2.1)	4.80 (2.4)	4.20 (2.3)

**Table 2:** Descriptive statistics for stress at work, mental strain, time pressure, and physical and mental exhaustion.

	Very Seldom or Never	Rather Seldom	Sometimes	Rather Often	Very Often or Always
Do you experience stress in your daily work?	0.9%	13.6%	59.1%	23.6%	2.7%
Do you experience mental strain at work?	0.9%	11.9%	55.0%	28.4%	3.7%
Are you under a lot of time-pressure at work?	1.9%	13.0%	42.6%	32.4%	10.2%
Are you physically exhausted at the end of the workday?	12.7%	24.5%	42.7%	16.4%	3.6%
Are you mentally exhausted at the end of the workday?	4.5%	18.2%	43.6%	28.2%	5.5%

**Table 3:** Do you get enough sleep?

	Yes, Five to Seven Nights per Week	Yes, Three to Four Nights per Week	No, Only Two Nights or Less per Week
Total	56.4%	31.8%	11.8%
Age (years):			
31–40	33.3%	50.0%	16.7%
41–50	57.9%	28.9%	13.2%
51–60	28.9%	32.1%	8.9%
>60	75.0%	-	25.0%
Seniority in a managerial position (years)			
<6	51.7%	34.5%	13.8%
6–10	65.0%	30.0%	5.0%
11–15	50.0%	38.5%	11.5%
>15	60.0%	25.7%	14.3%
Number of full-time positions in nursing:			
1–6	50.0%	33.3%	16.7%
7–10	50.0%	38.5%	11.5%
11–15	72.0%	24.0%	4.0%
>15	51.9%	40.7%	7.4%
Working hours per day			
≤8	60.0%	31.1%	8.9%
9	48.9%	38.3%	12.8%
10	75.0%	16.7%	8.3%
11	40.0%	20.0%	40.0%

**Table 4:** Results of the correlation between pain/discomfort in the neck and neck area, shoulder and shoulder area, and lower back and stressful factors in the working environment and lack of adequate sleep.

Scale	1	2	3	4	5	6	7	8	9
1. Pain in neck and neck area	-	0.707 *	0.463 *	0.126	0.220 *	0.139	0.428 *	0.317 *	−0.314 *
2. Pain in shoulder and shoulder area		-	0.552 *	0.083	0.148	0.140	0.459 *	0.300 *	−0.334 *
3. Pain in lower back			-	0.186	0.094	0.123	0.302 *	0.114	−0.283 *
4. Do you experience stress in your daily work?				-	0.626 *	0.522 *	0.405 *	0.476 *	−0.362 *
5. Do you experience mental strain at work?					-	0.484 *	0.456 *	0.630 *	−0.275 *
6. Are you under a lot of time pressure at work?						-	0.448 *	0.523 *	−0.327 *
7. Are you physically exhausted at the end of the workday?							-	0.534 *	−0.384 *
8. Are you mentally exhausted at the end of the workday?								-	−0.345 *
9. Do you get enough sleep?									-

* p < 0.05 (two-tailed).
